# Supplementary material for: Embryonic stem cells maintain high origin activity and slow forks to coordinate replication with cell cycle progression
Source: EMBO Rep. 2024 Jul 25;25(9):6. doi: 10.1038/s44319-024-00207-5 (PMC11387781; doi:10.1038/s44319-024-00207-5)
Supplement: Supplementary file 1 — Appendix [file 44319_2024_207_MOESM1_ESM.pdf]

Table of contents:

Appendix Figure S1
containing images related to Fig. EV1B (A),
Fig. 2E and Fig. 4B (B), Fig. 3D (C) and Fig. 4G (D) …….1

Legend for Appendix Figure S1 ……………………….2

Appendix Figure S1

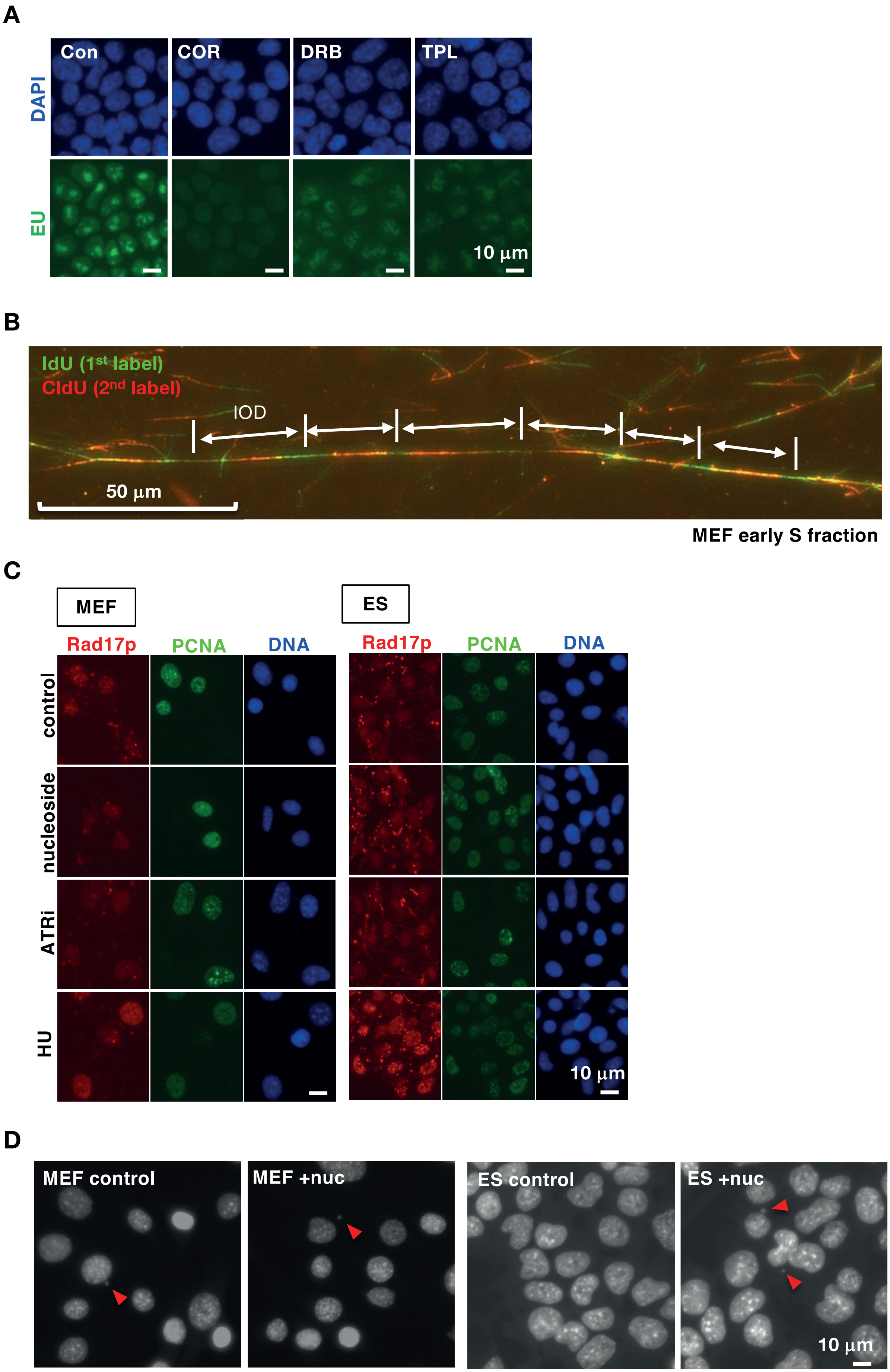

Appendix Figure S1.

Appendix Figure S1.

A (related to Fig. EV1B) DAPI and EU-Click images of ES cells treated with transcription inihibitors for 2.5 hours. COR, 50 μM cordycepin; DRB, 25 μM dichloro-ribofuranosylbenzimidazole; TPL, 1 μM triptolide.

B (related to Fig. 2E and Fig. 4B) An example of DNA fiber used to score inter-origin distances (IODs). The center of an IdU (first label)–stained region is defined as an initiation site, and distances between the two adjacent initiation sites are scored as IODs.

C (related to Fig. 3D) Examples of MEF and ES cell images treated with or without nucleosides, ATRi (1 μM for 2 hours), or HU (1 mM for 2 hours), fixed and stained with anti-pRad17 and anti-PCNA antibodies and DAPI.

D (related to Fig. 4G) Examples of MEF and ES cell images presenting micronuclei (indicated with red arrowheads), treated with or without nucleosides (48 hours) and stained with DAPI.
