# Supplementary figures and images for: Embryonic stem cells maintain high origin activity and slow forks to coordinate replication with cell cycle progression
Source: EMBO Rep. 2024 Jul 25;25(9):6. doi: 10.1038/s44319-024-00207-5 (PMC11387781; doi:10.1038/s44319-024-00207-5)

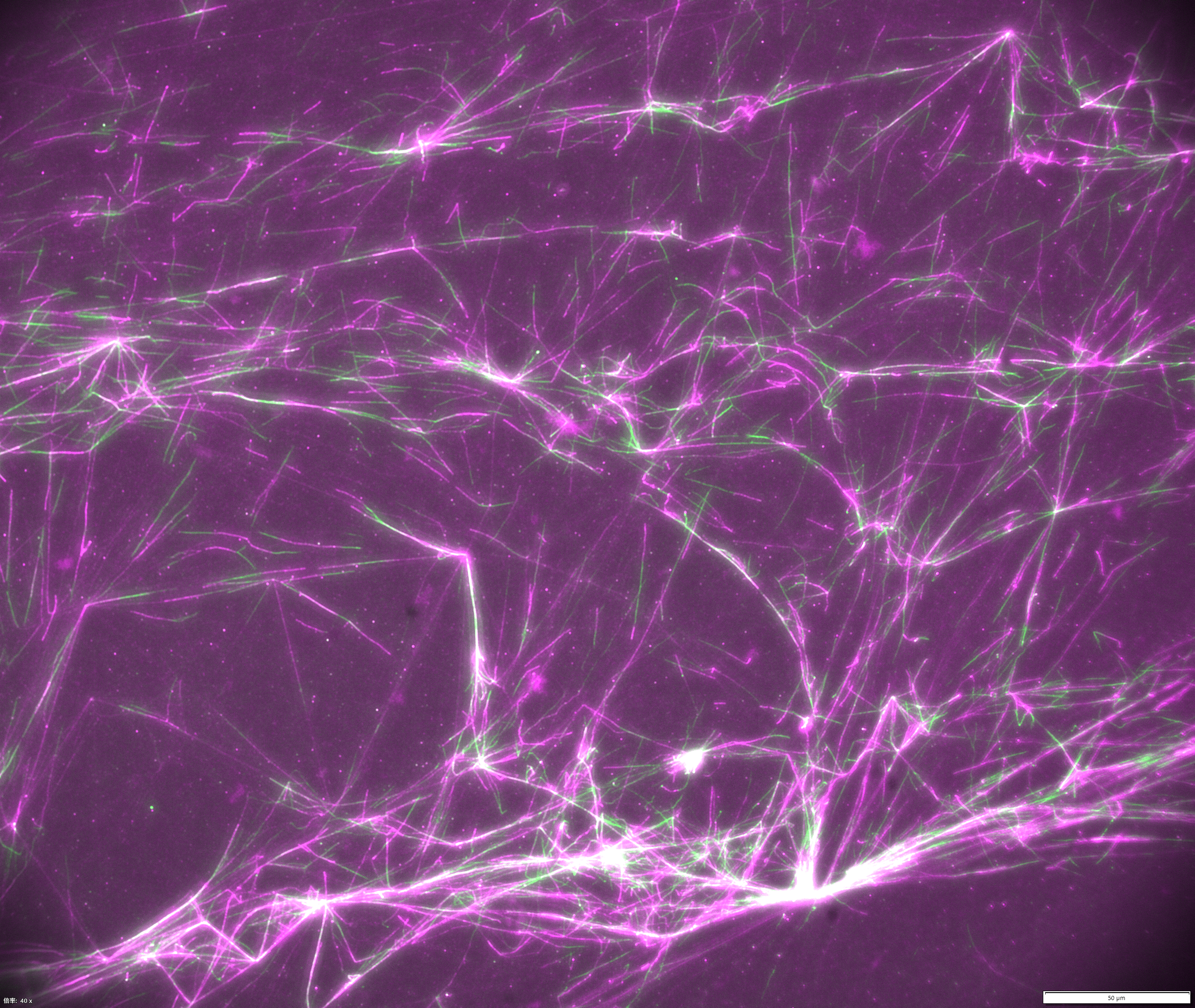

Supplement: Supplementary file 4 — Source data Fig. 2 [file 44319_2024_207_MOESM4_ESM.zip › EMBOR-2024-58881_SourceDataForFigure2/EMBOR-2024-58881_SourceDataFor2A/EMBOR-2024-58881_SourceDataFor2Afiber example4.tif]

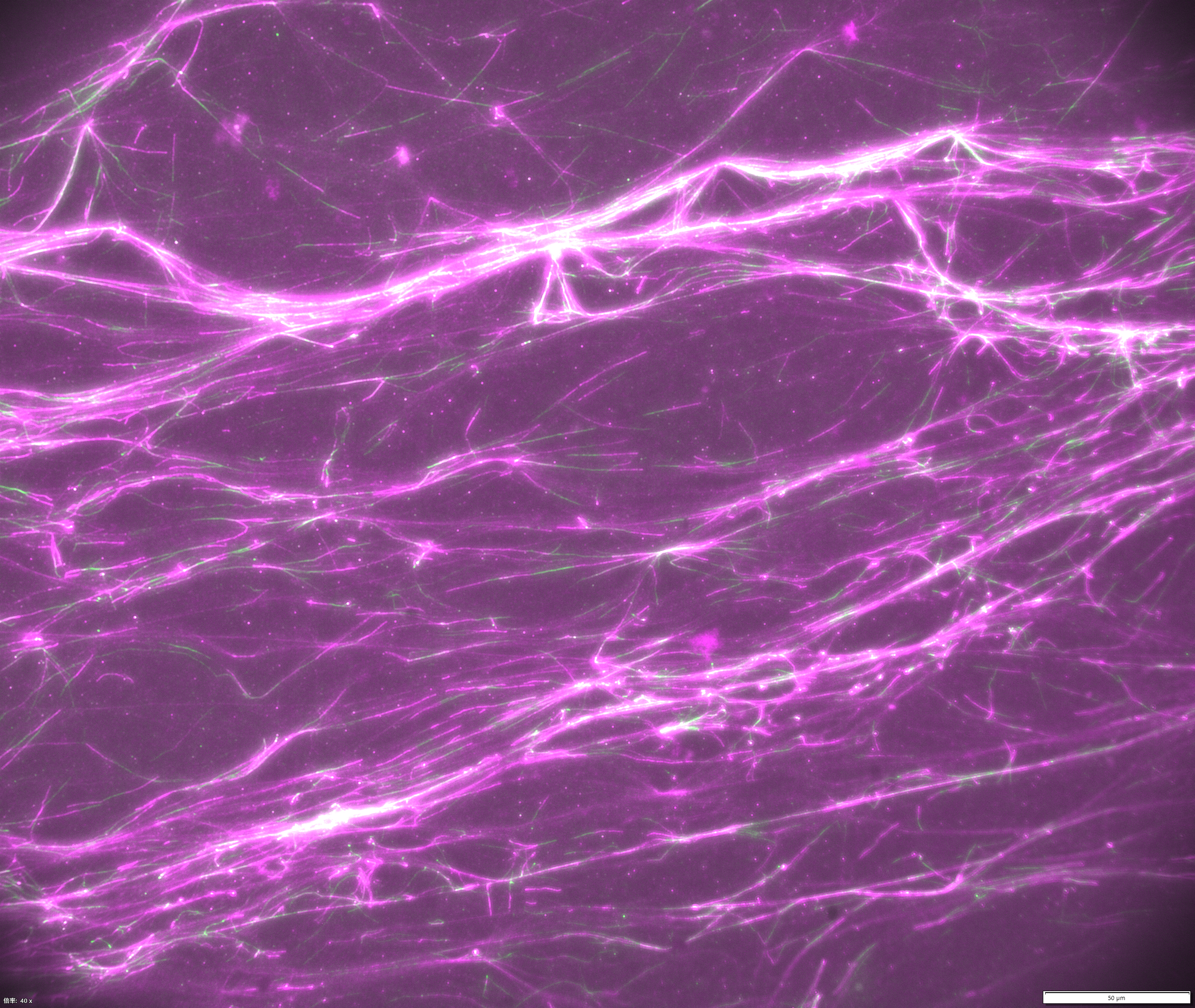

Supplement: Supplementary file 4 — Source data Fig. 2 [file 44319_2024_207_MOESM4_ESM.zip › EMBOR-2024-58881_SourceDataForFigure2/EMBOR-2024-58881_SourceDataFor2A/EMBOR-2024-58881_SourceDataFor2Afiber example5.tif]

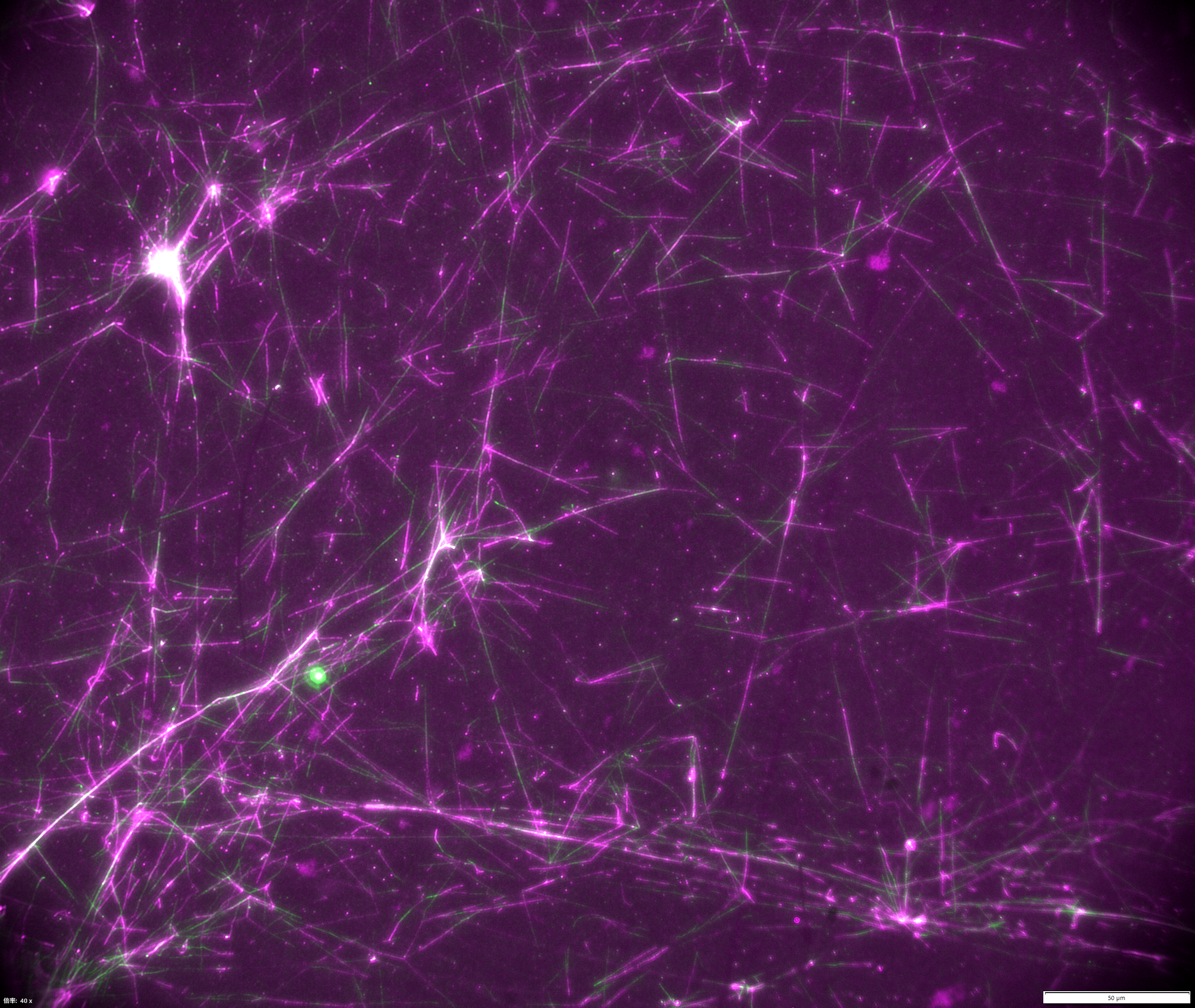

Supplement: Supplementary file 4 — Source data Fig. 2 [file 44319_2024_207_MOESM4_ESM.zip › EMBOR-2024-58881_SourceDataForFigure2/EMBOR-2024-58881_SourceDataFor2A/EMBOR-2024-58881_SourceDataFor2Afiber example1.tif]

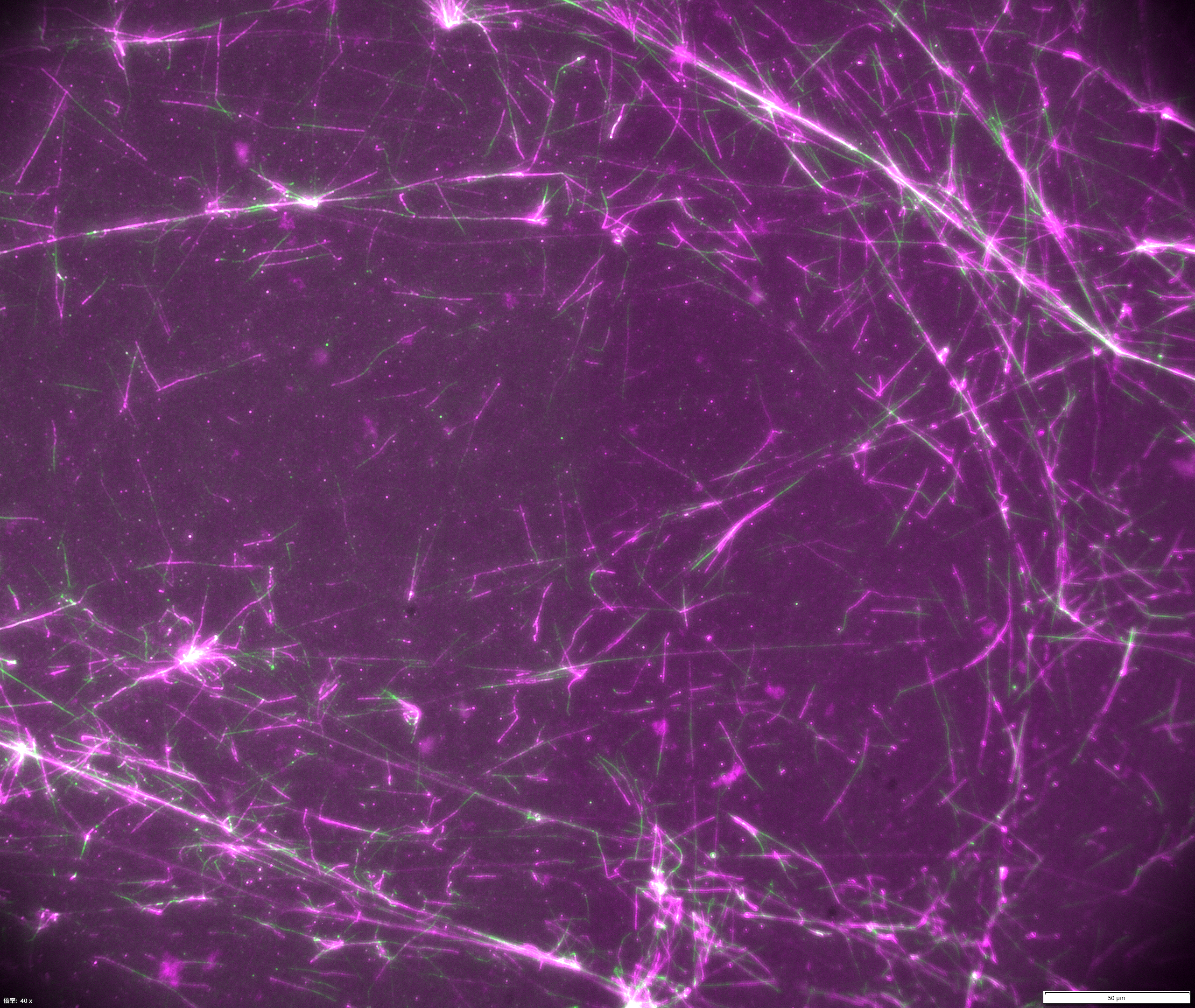

Supplement: Supplementary file 4 — Source data Fig. 2 [file 44319_2024_207_MOESM4_ESM.zip › EMBOR-2024-58881_SourceDataForFigure2/EMBOR-2024-58881_SourceDataFor2A/EMBOR-2024-58881_SourceDataFor2Afiber example2.tif]

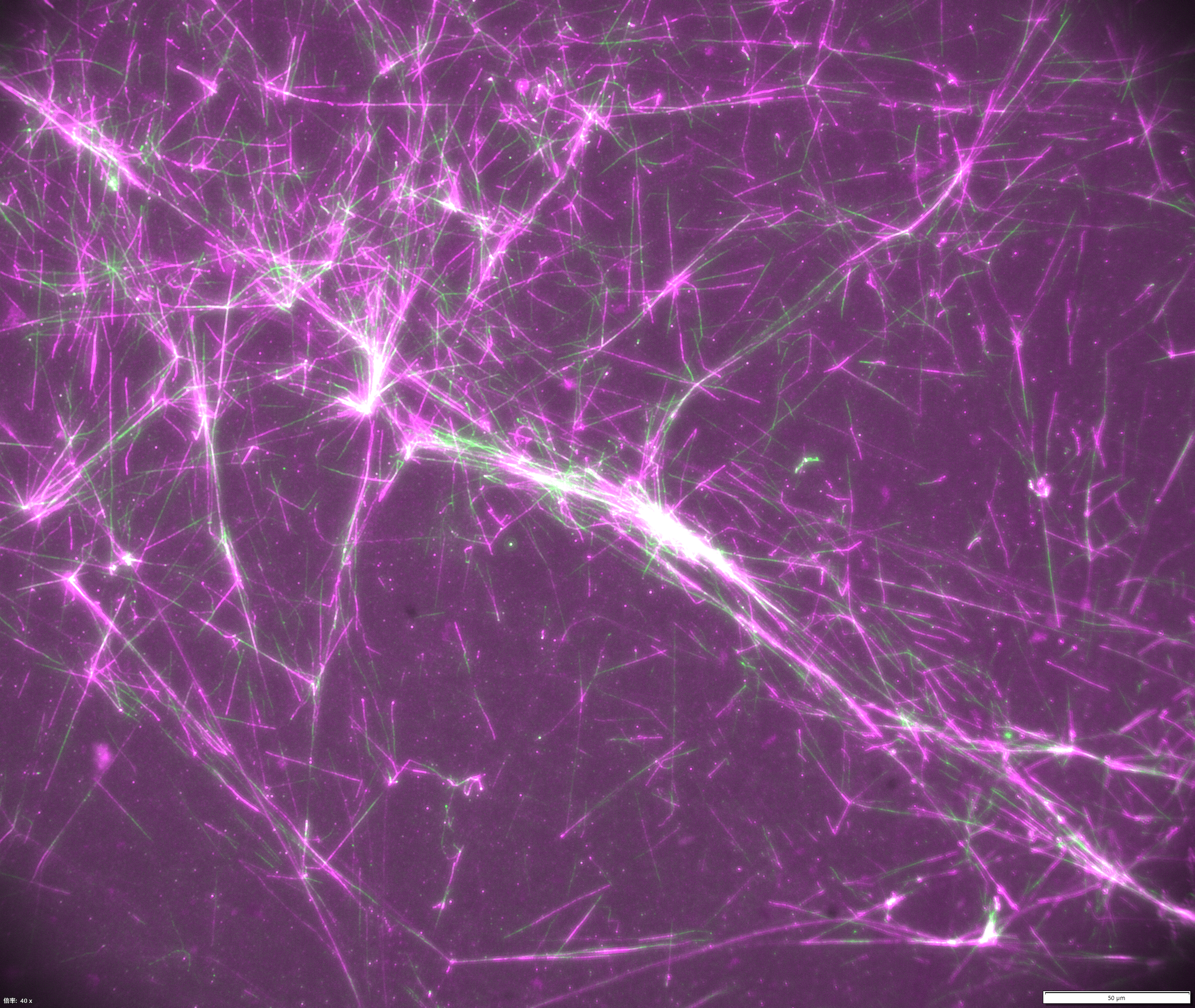

Supplement: Supplementary file 4 — Source data Fig. 2 [file 44319_2024_207_MOESM4_ESM.zip › EMBOR-2024-58881_SourceDataForFigure2/EMBOR-2024-58881_SourceDataFor2A/EMBOR-2024-58881_SourceDataFor2Afiber example3.tif]

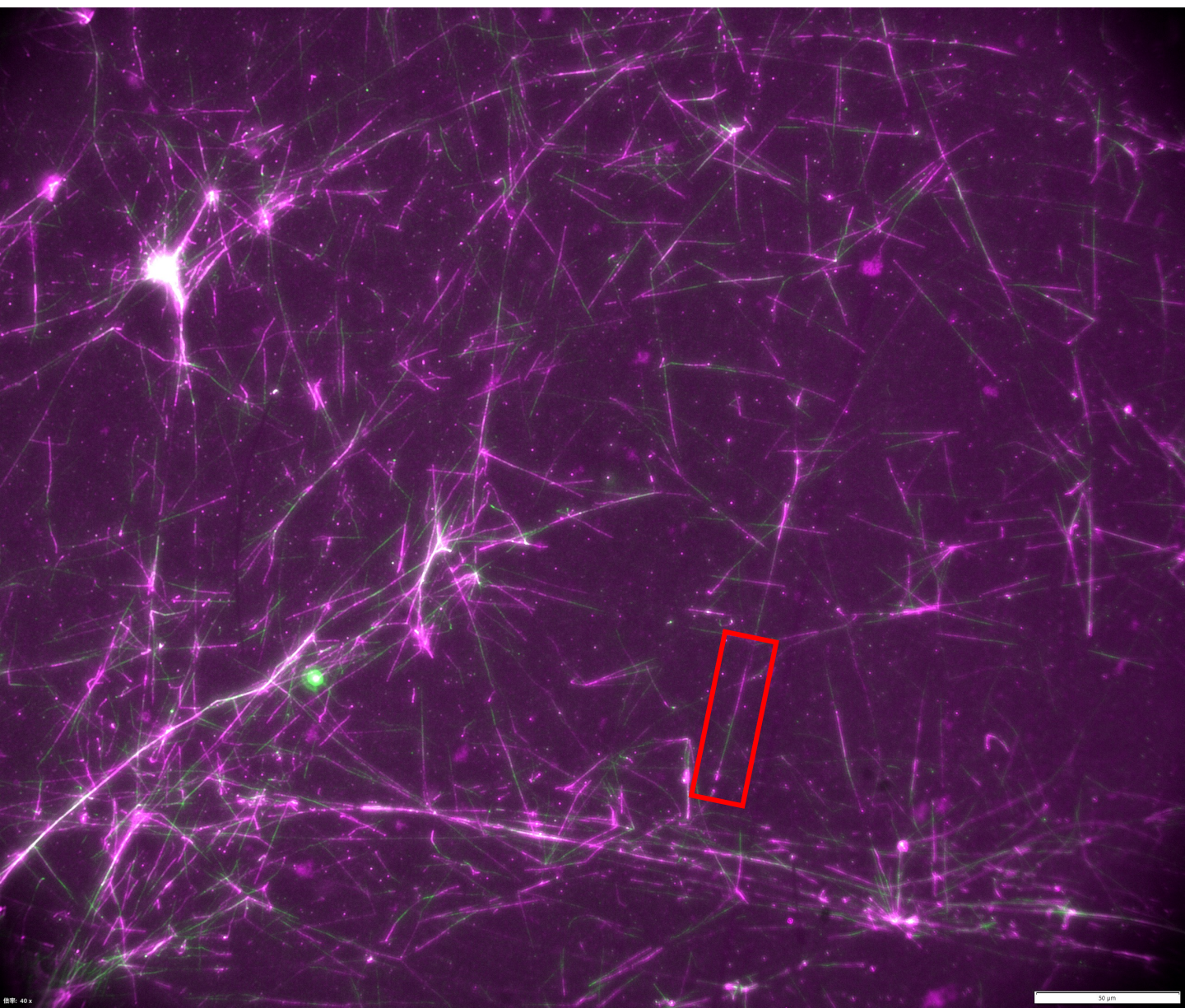

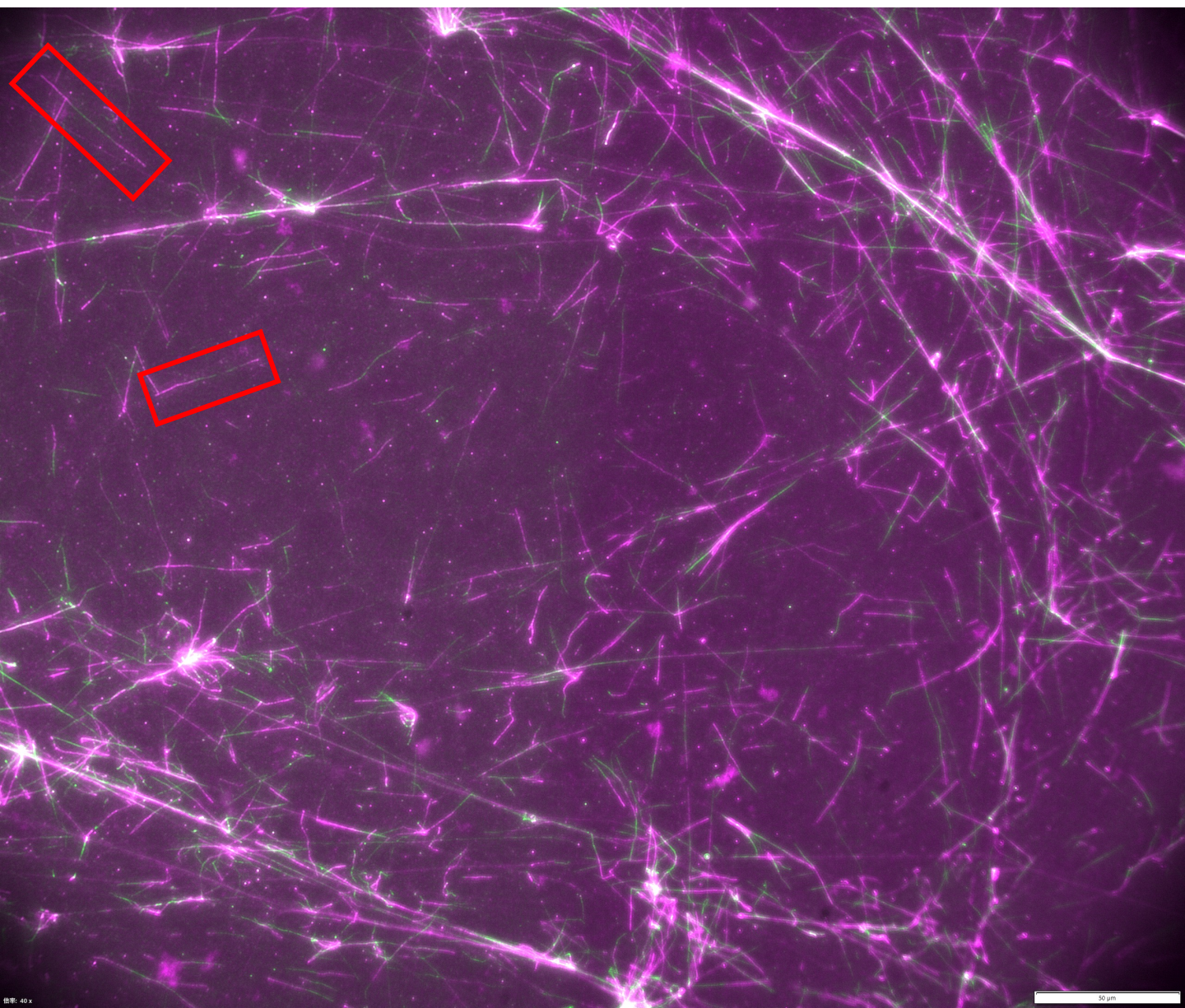

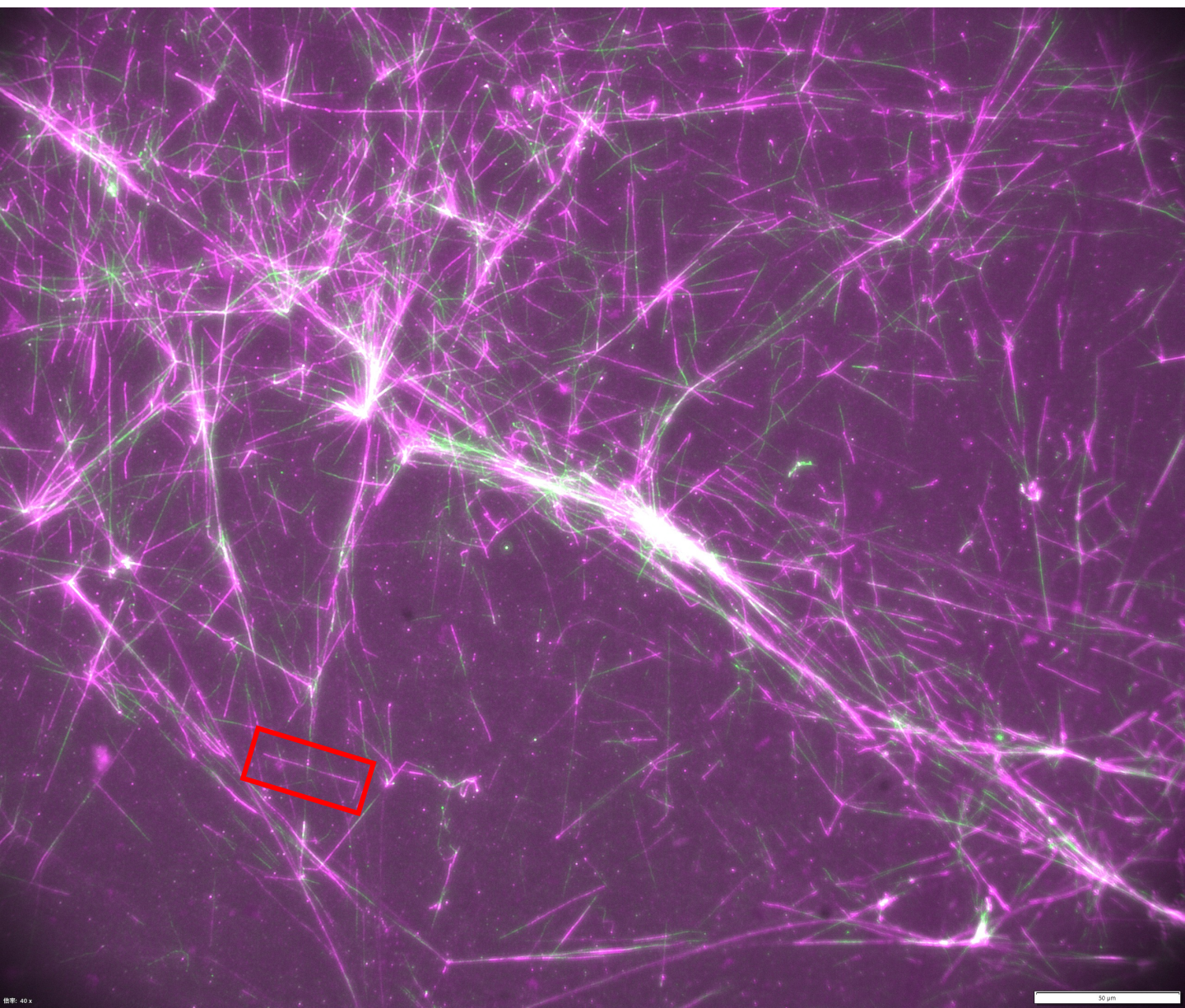

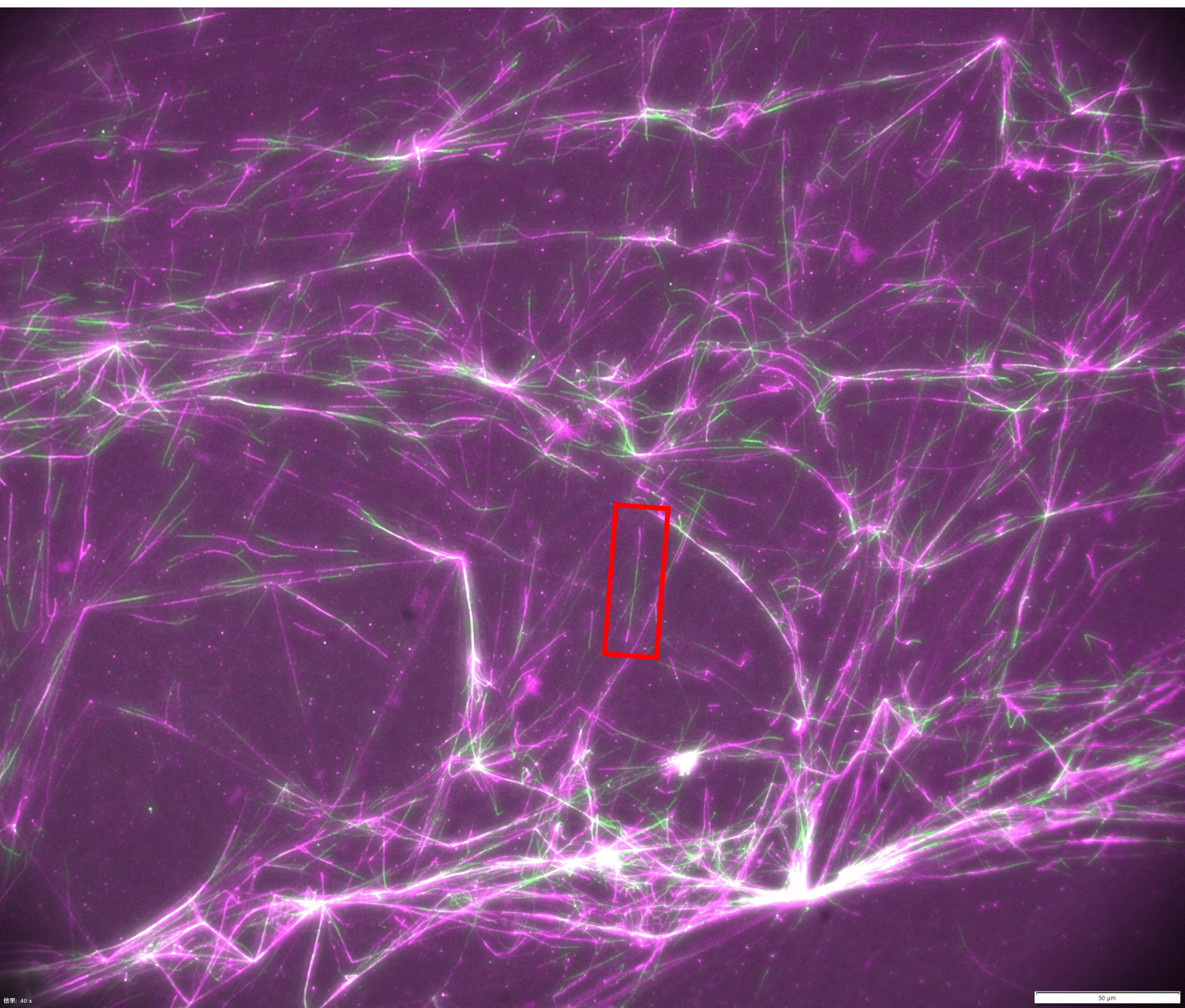

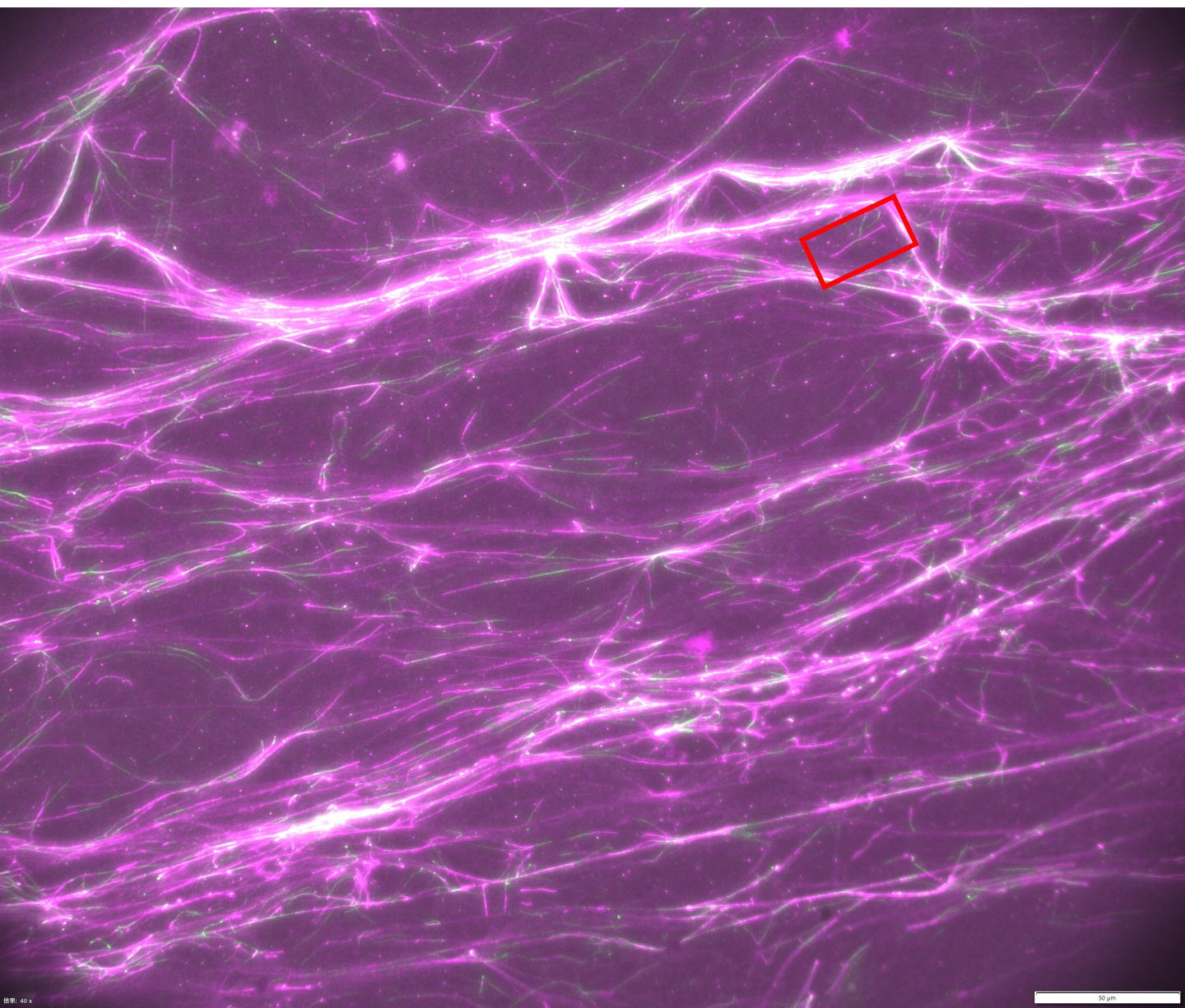

Supplement: Supplementary file 4 — Source data Fig. 2 [file 44319_2024_207_MOESM4_ESM.zip › EMBOR-2024-58881_SourceDataForFigure2/EMBOR-2024-58881_SourceDataFor2A/EMBOR-2024-58881_SourceDataFor2Afiber example.pdf]

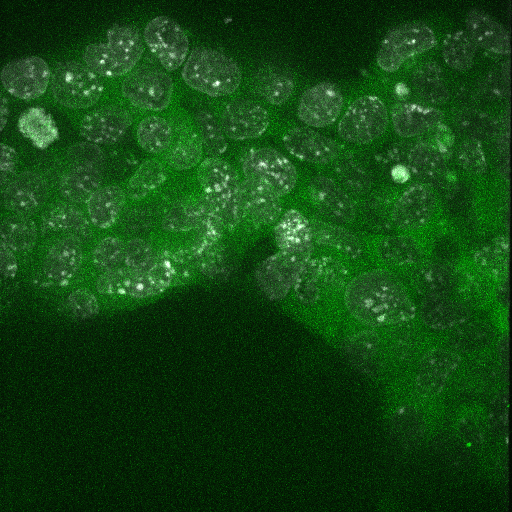

Supplement: Supplementary file 6 — Source data Fig. 4 [file 44319_2024_207_MOESM6_ESM.zip › EMBOR-2024-58881_SourceDataForFigure4/EMBOR-2024-58881_SourceDataFor4E/EMBOR-2024-58881_SourceDataFor4EClean _1.tif]

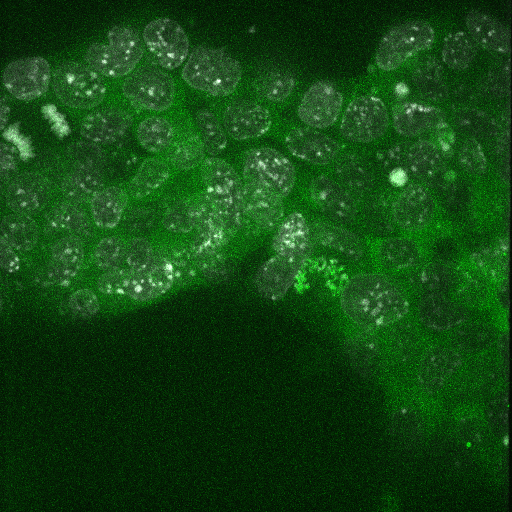

Supplement: Supplementary file 6 — Source data Fig. 4 [file 44319_2024_207_MOESM6_ESM.zip › EMBOR-2024-58881_SourceDataForFigure4/EMBOR-2024-58881_SourceDataFor4E/EMBOR-2024-58881_SourceDataFor4EClean _2.tif]

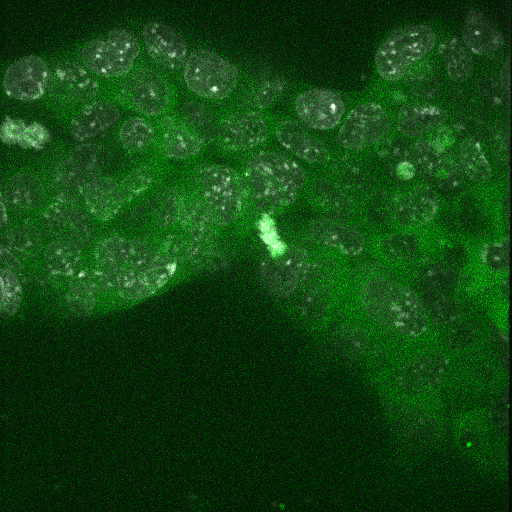

Supplement: Supplementary file 6 — Source data Fig. 4 [file 44319_2024_207_MOESM6_ESM.zip › EMBOR-2024-58881_SourceDataForFigure4/EMBOR-2024-58881_SourceDataFor4E/EMBOR-2024-58881_SourceDataFor4EUFB_1.tif]

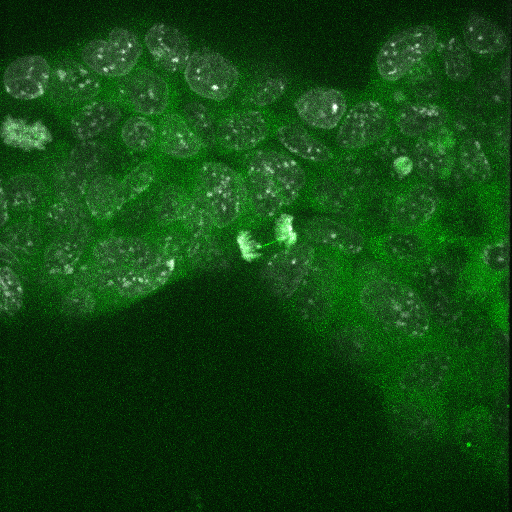

Supplement: Supplementary file 6 — Source data Fig. 4 [file 44319_2024_207_MOESM6_ESM.zip › EMBOR-2024-58881_SourceDataForFigure4/EMBOR-2024-58881_SourceDataFor4E/EMBOR-2024-58881_SourceDataFor4EUFB_2.tif]

UFB

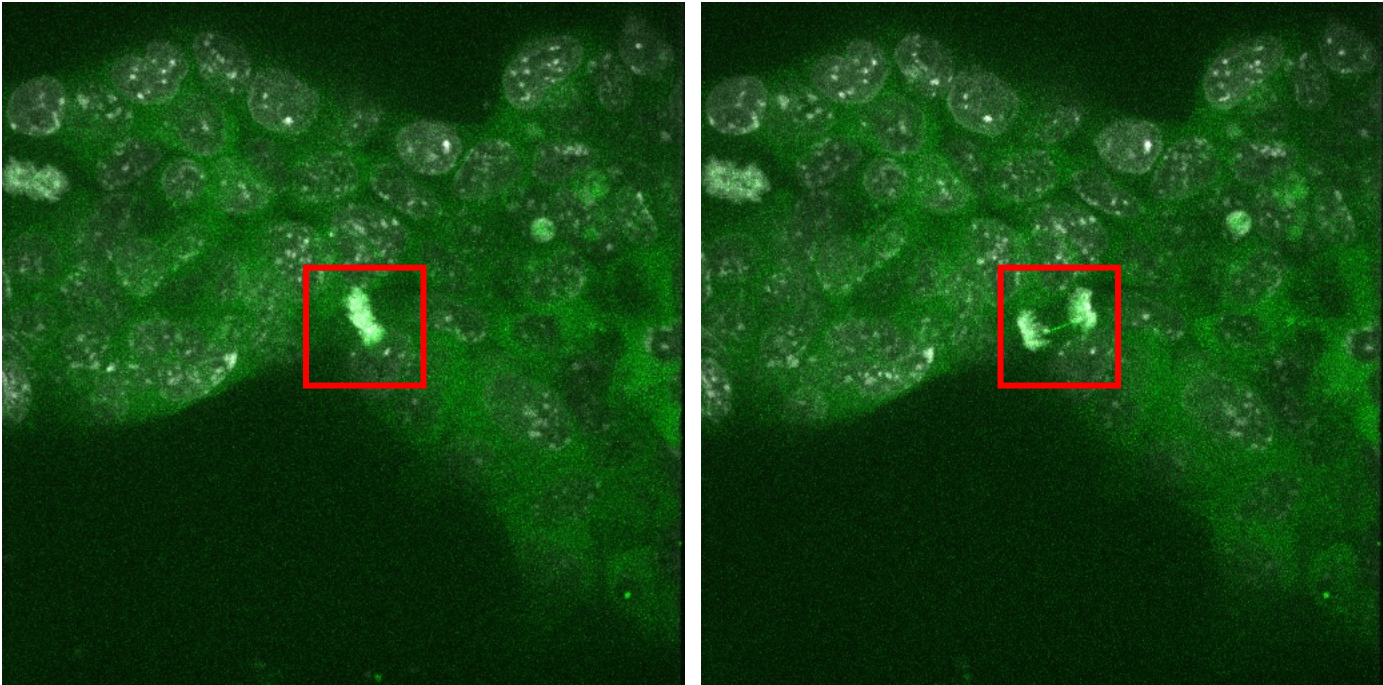

clean segregation

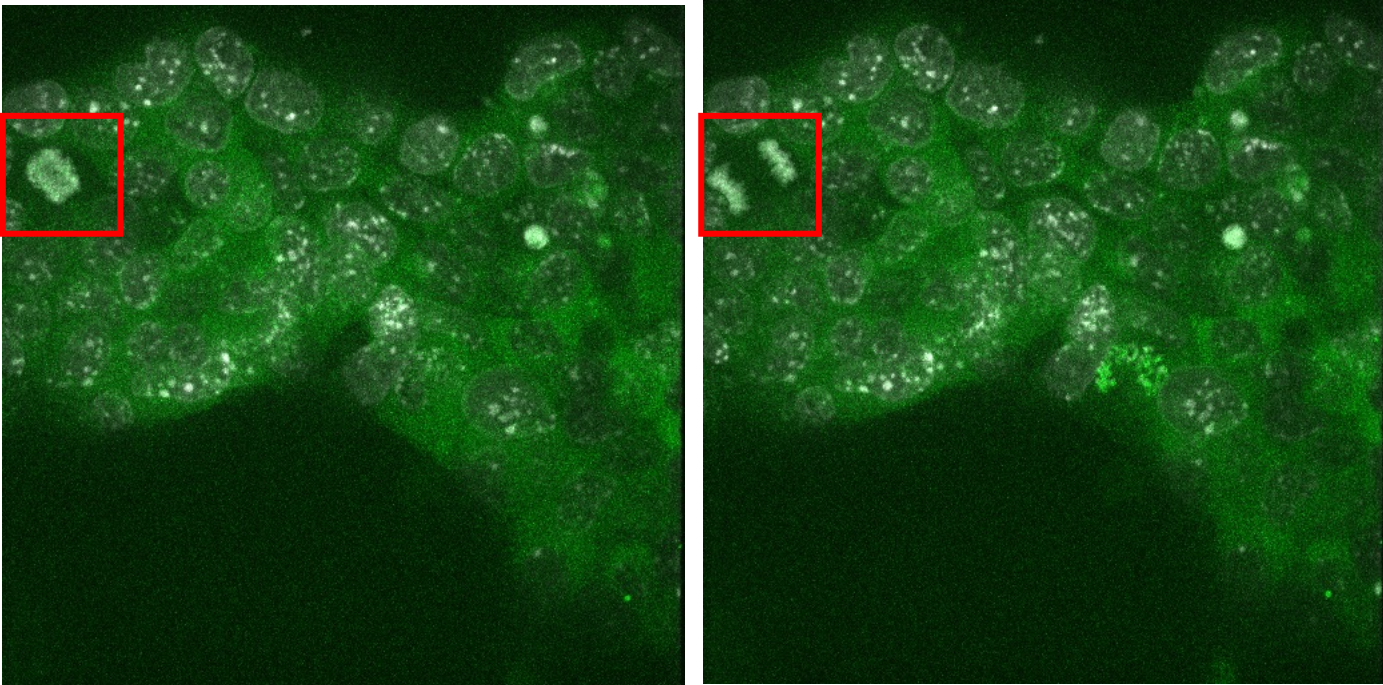

Supplement: Supplementary file 6 — Source data Fig. 4 [file 44319_2024_207_MOESM6_ESM.zip › EMBOR-2024-58881_SourceDataForFigure4/EMBOR-2024-58881_SourceDataFor4E/EMBOR-2024-58881_SourceDataFor4EUFB.pdf]

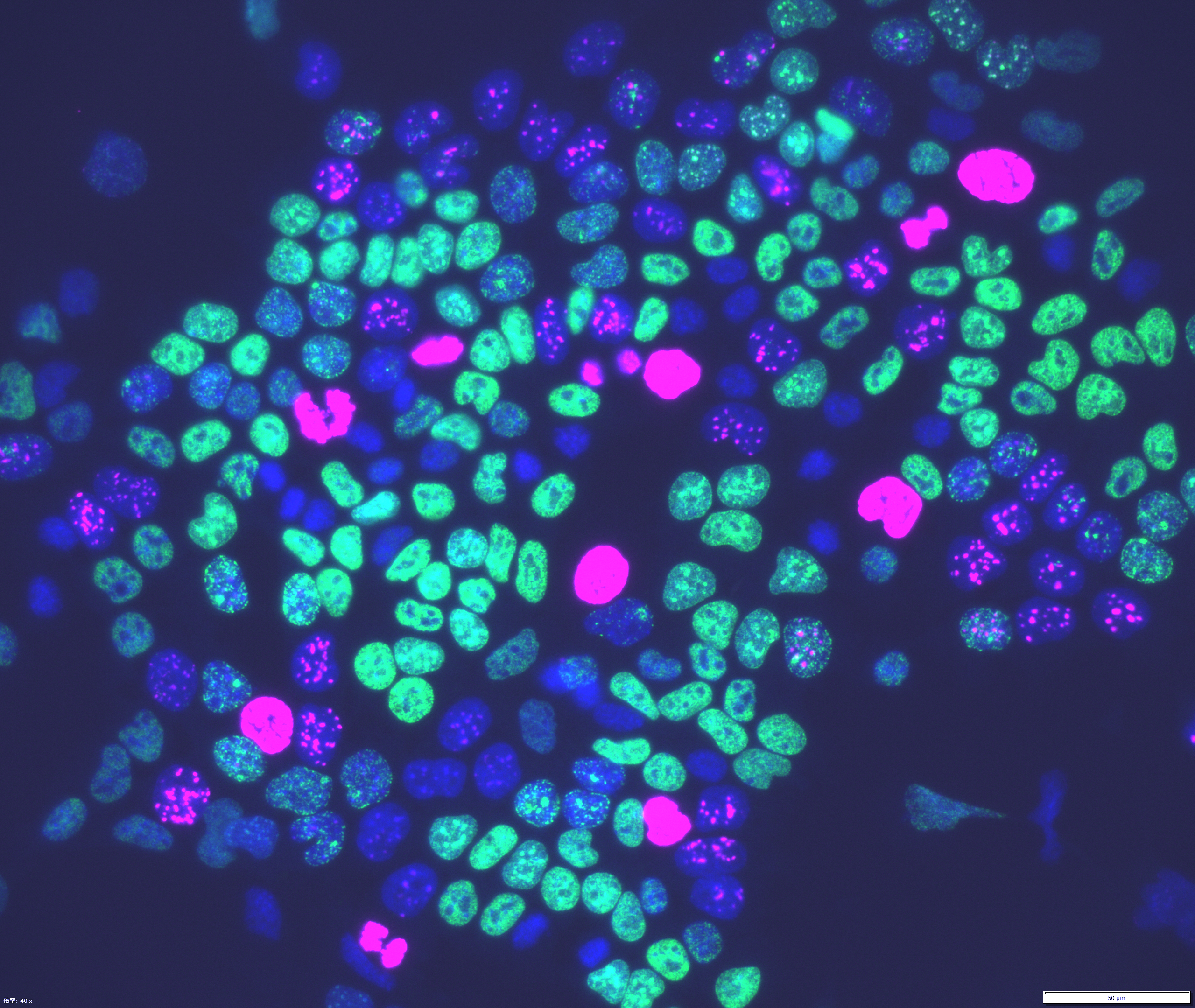

Supplement: Supplementary file 6 — Source data Fig. 4 [file 44319_2024_207_MOESM6_ESM.zip › EMBOR-2024-58881_SourceDataForFigure4/EMBOR-2024-58881_SourceDataFor4C/EMBOR-2024-58881_SourceDataFor4CES-con.tif]

Control

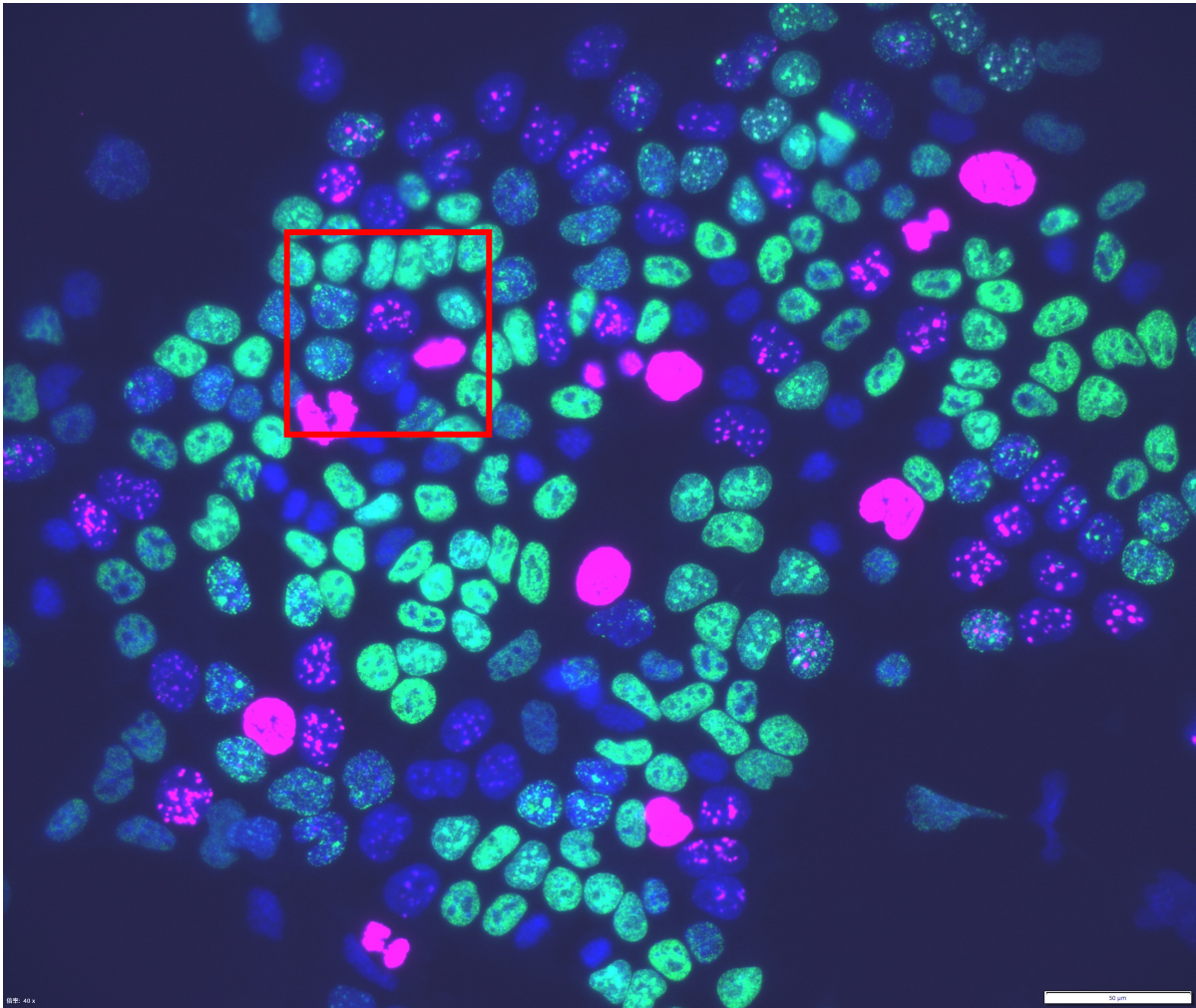

nucleoside

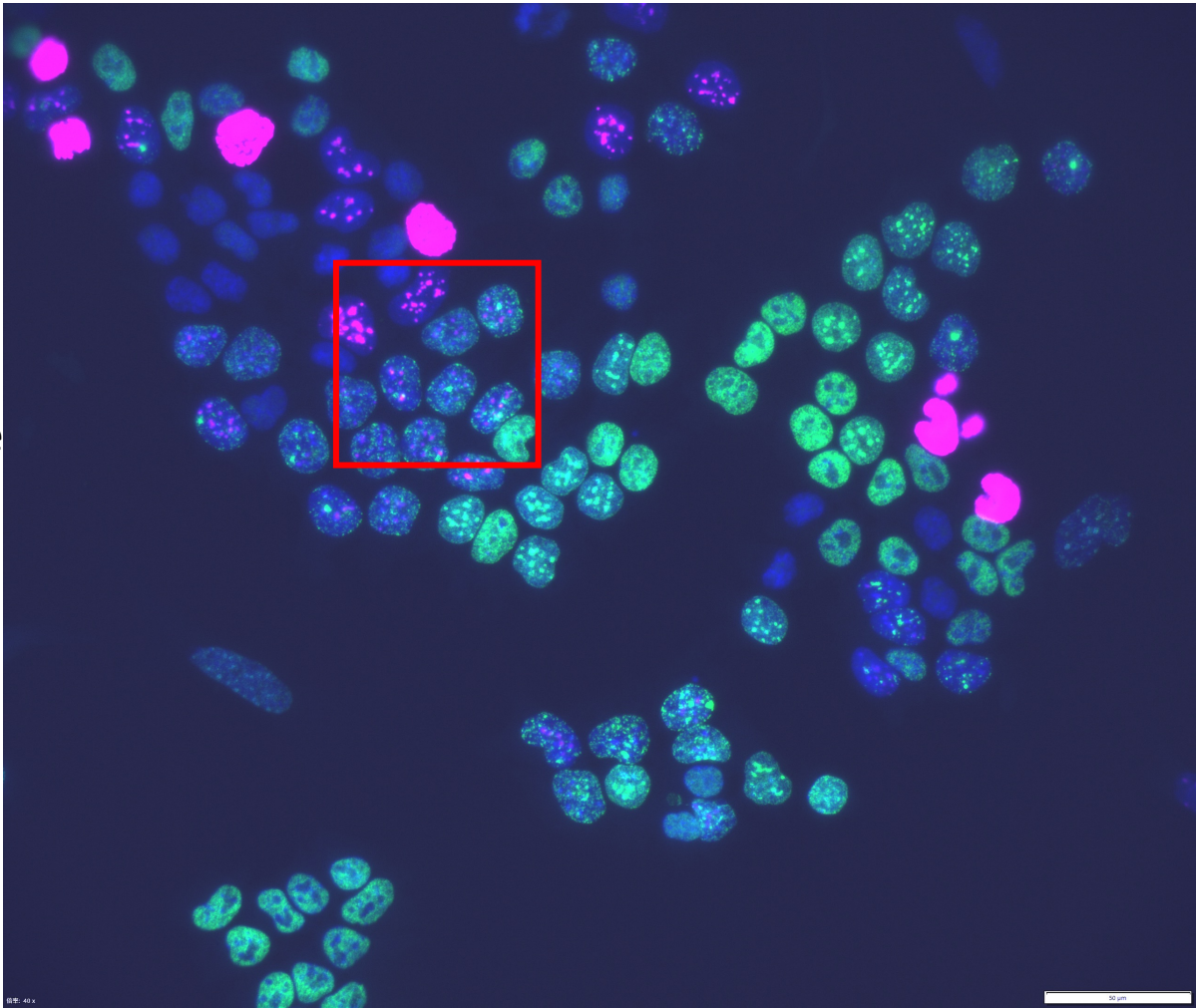

Supplement: Supplementary file 6 — Source data Fig. 4 [file 44319_2024_207_MOESM6_ESM.zip › EMBOR-2024-58881_SourceDataForFigure4/EMBOR-2024-58881_SourceDataFor4C/EMBOR-2024-58881_SourceDataFor4CEdU H3S10p.pdf]

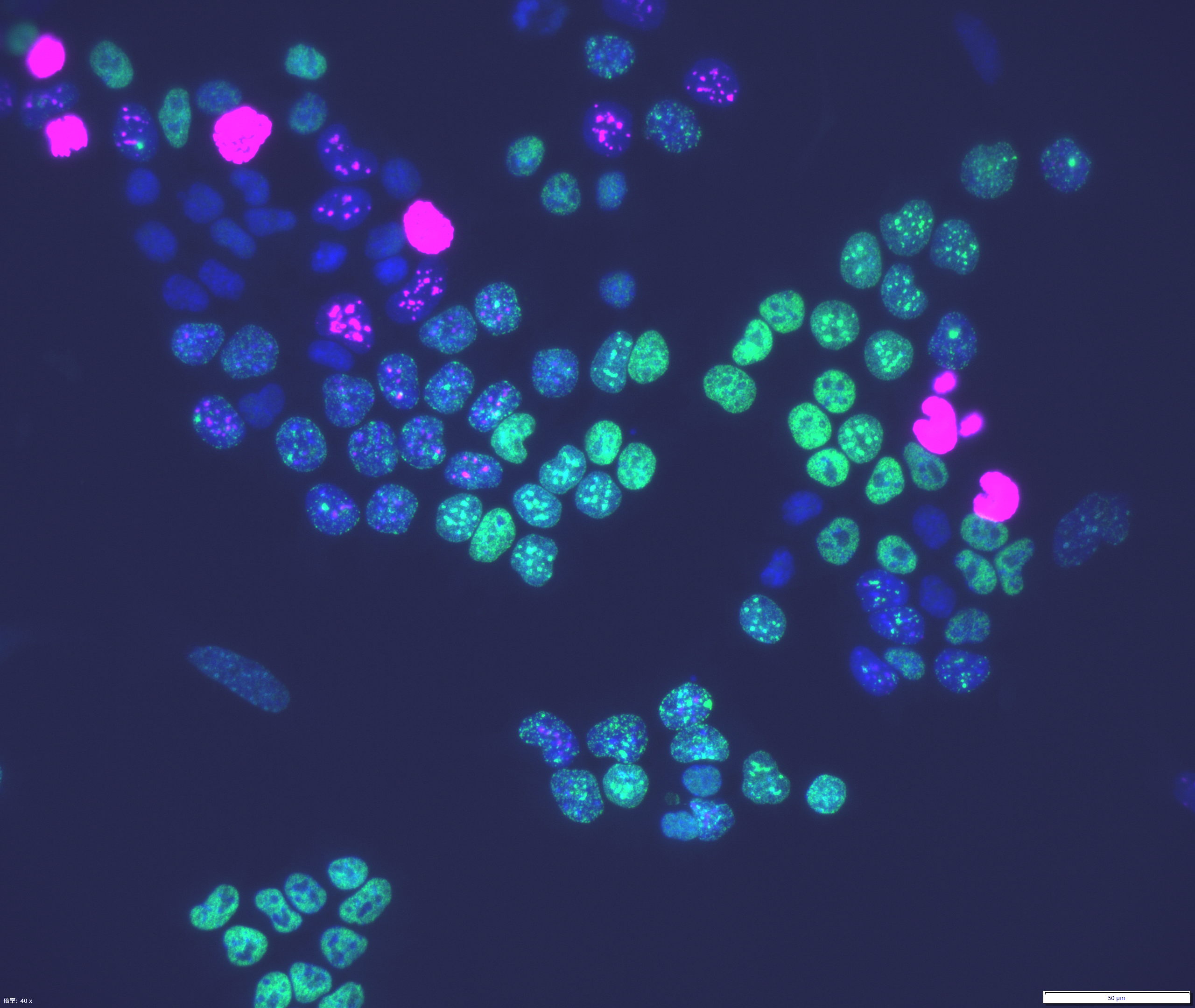

Supplement: Supplementary file 6 — Source data Fig. 4 [file 44319_2024_207_MOESM6_ESM.zip › EMBOR-2024-58881_SourceDataForFigure4/EMBOR-2024-58881_SourceDataFor4C/EMBOR-2024-58881_SourceDataFor4CES-nuc.tif]
