# Supplementary material for: Embryonic stem cells maintain high origin activity and slow forks to coordinate replication with cell cycle progression
Source: EMBO Rep. 2024 Jul 25;25(9):6. doi: 10.1038/s44319-024-00207-5 (PMC11387781; doi:10.1038/s44319-024-00207-5)
Supplement: Supplementary file 7 — Expanded View Figures [file 44319_2024_207_MOESM7_ESM.pdf]

Expanded View Figures

Figure EV1. Effects of culturing media, fixation procedures, transcription, and reactive oxygen species (ROS) on replication fork speed measured by DNA fiber assay. ▶

(**A**) MEFs were grown in the usual (MEF) or ES media for 48 h and were subjected to DNA fiber assay. In both repeated experiments (exp1 and 2), the fork speed of MEFs in ES media was not slower compared to when they were grown in the usual media. (**B**) Transcription was inhibited with 50 µM cordycepin (COR), 25 µM dichloro-ribofuranosylbenzimidazole (DRB), and 1 µM triptolide (TPL) for 2.5 h in ES cells, and their effects were evaluated by EU-Click staining ("Methods", Appendix Fig. S1A). EU intensities per nucleus were scored and plotted. The numbers of scored cells are indicated in red. (**C**) Fork speeds in ES cells treated with transcription inhibitors as in (**B**). (**D**) The effect of reactive oxygen species (ROS) was assessed by adding a ROS scavenger, N-acetyl-l-cysteine (NAC). Low doses of HU are known to increase ROS levels. As expected, NAC reversed the fork slowdown observed with 10 µM HU, but NAC alone does not increase fork speed in ES cells, indicating that fork slowdown in ES cells is not due to ROS. (**E**) Fork speed measurements were done using ES cells that were collected after pulse-labeling and cell pellets were resuspended in PBS, 70% ethanol (EtOH) or 3:1 methanol/acetic acid (MeOH). Fixed cells were kept at 4 degrees overnight (PBS, MeOH) or at -30 degrees for 1day or 5 days (EtOH). (**F**) Replication fork speed of N2A, mB and RPE cells in early (E), early–mid (EM), mid–late (ML) and late (L) fractions (as done in Fig. 1D). (**G**) ES cells were either cultured in a normal condition (control) or without LIF for 3 days (-LIF) and were pelleted to carry out quantitative PCR to compare expression levels of genes associated with pluripotency (left). Gene expression levels were determined relative to *mGAPDH* which were then compared relative to control samples. (middle) Immunofluorescent staining for Oct4 and DNA (DAPI) in cells grown in normal media or media without LIF. (right) Cell morphology in control and -LIF conditions. Numbers of scored forks are indicated in red for (**A**, **C**–**F**). Data in (**G**) are presented as mean +/−SD ($n = 3$ biological repeats, each with $n = 2$ technical repeats). NS, not significant ($P \geqq 0.05$), *$P < 0.05$, ***$P < 0.001$, ****$P < 0.0001$, Mann–Whitney tests (**A**, **B**, **D**), Kruskal–Wallis tests (**C**, **E**, **F**) and two-tailed *t* test (**G**). Exact *P* values are also indicated. Data from a representative experiment is shown ($n = 2$ independent experiments) for (**A**–**F**).

 © The Author(s)

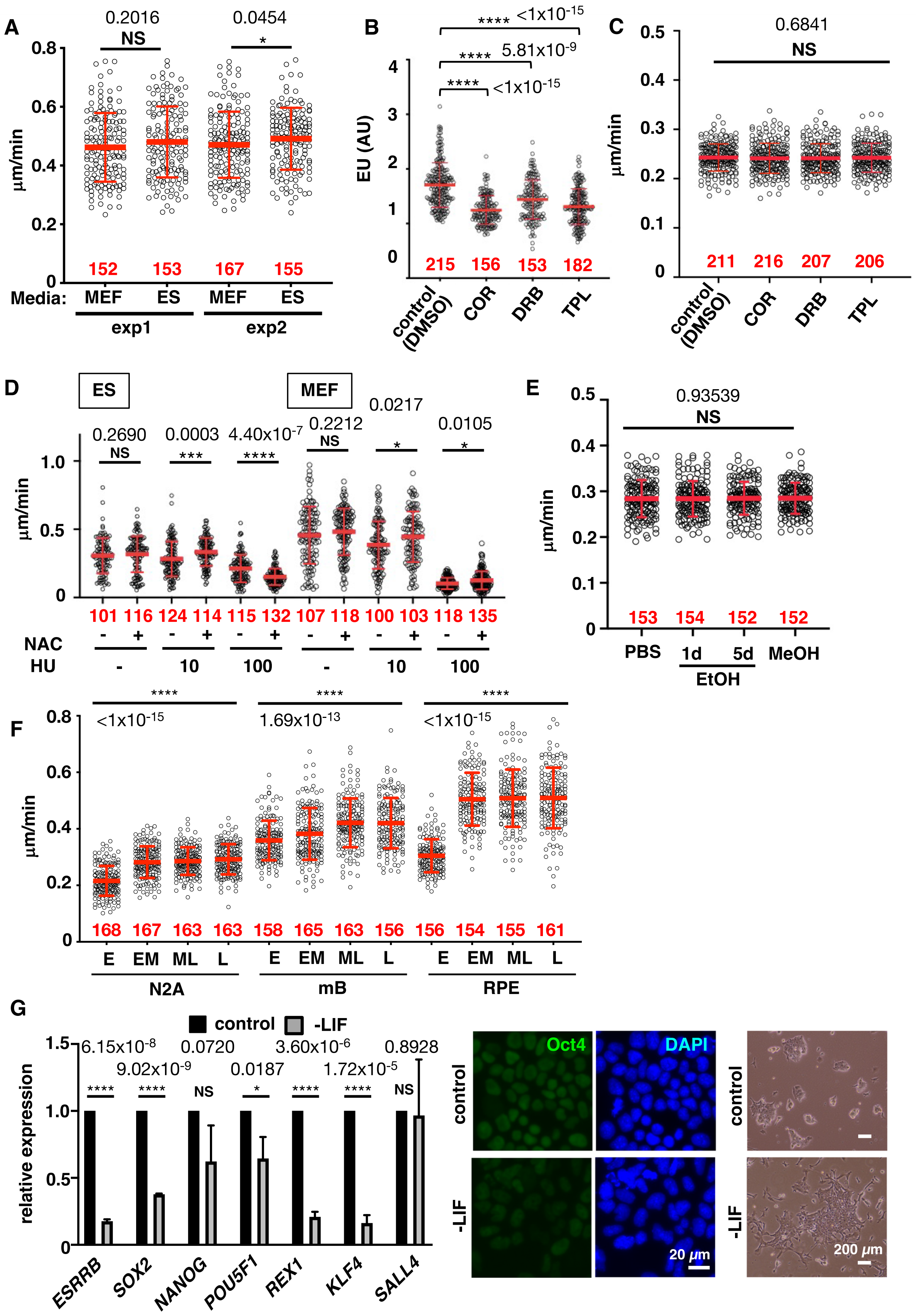
A
0.2016
NS
0.0454
*
μm/min
0.8
0.6
0.4
0.2
0
152 153 167 155
Media: MEF ES MEF ES
exp1
exp2
B
**** <1x10-15
**** 5.81x10-9
**** <1x10-15
EU (AU)
4
3
2
1
0
215 156 153 182
control (DMSO)
COR
DRB
TPL
C
0.6841
NS
μm/min
0.5
0.4
0.3
0.2
0.1
0
211 216 207 206
control (DMSO)
COR
DRB
TPL
D
ES
MEF
0.2690 NS
0.0003 ***
4.40x10-7 ****
0.2212 NS
0.0217 *
0.0105 *
μm/min
1.0
0.5
0
101 116 124 114 115 132 107 118 100 103 118 135
NAC - + - + - + - + - + - +
HU - 10 100 - 10 100
E
0.93539
NS
μm/min
0.5
0.4
0.3
0.2
0.1
0
153 154 152 152
PBS
1d 5d
EtOH
MeOH
F
**** <1x10-15
**** 1.69x10-13
**** <1x10-15
μm/min
0.8
0.6
0.4
0.2
0
168 167 163 163 158 165 163 156 156 154 155 161
E EM ML L E EM ML L E EM ML L
N2A
mB
RPE
G
control
-LIF
relative expression
1.5
1.0
0.5
0
6.15x10-8 ****
9.02x10-9 ****
0.0720 NS
0.0187 *
3.60x10-6 ****
1.72x10-5 ****
0.8928 NS
ESRRB
SOX2
NANOG
POU5F1
REX1
KLF4
SALL4
Oct4
DAPI
control
-LIF
20 μm
control
-LIF
200 μm

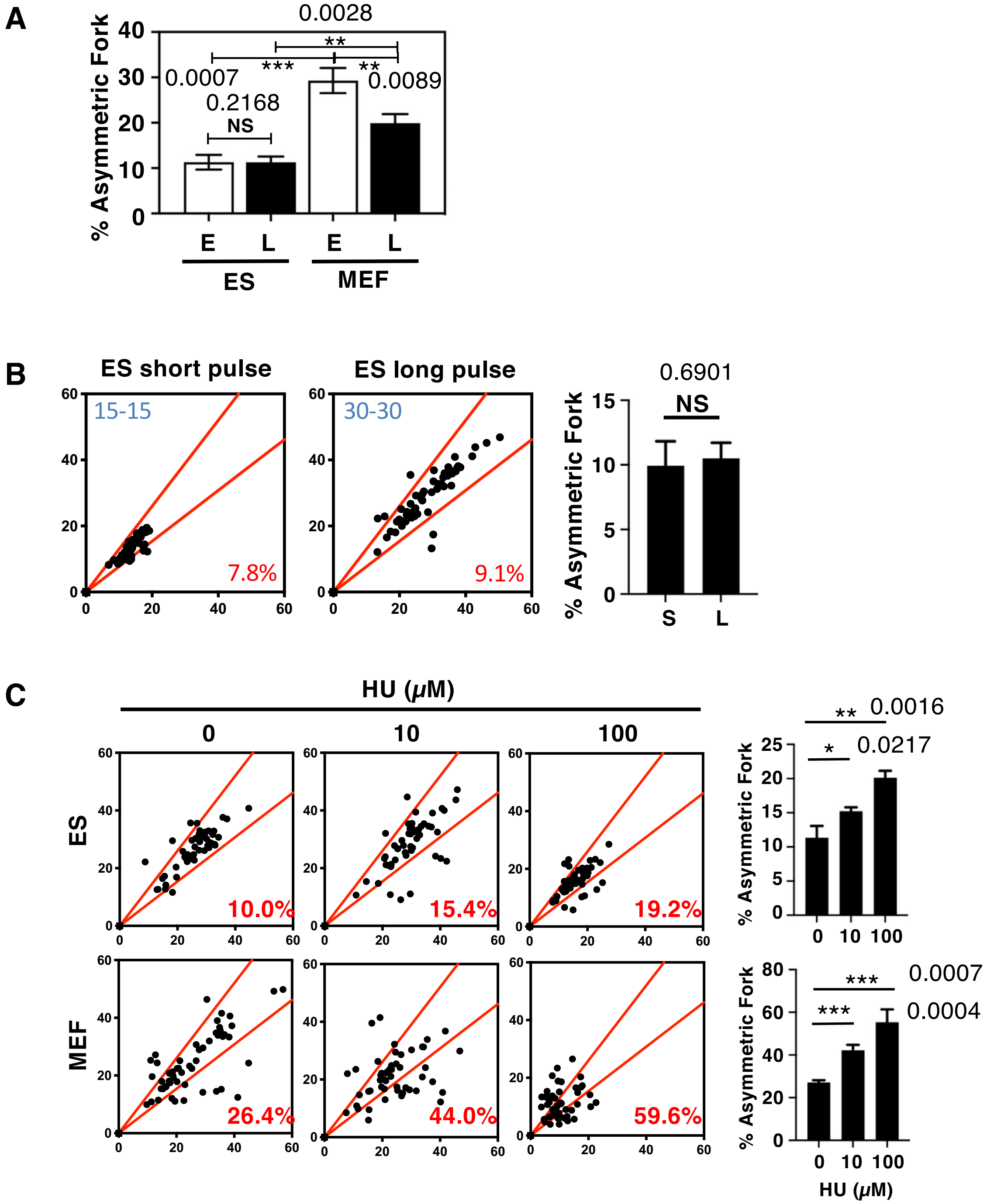

Figure EV2. Effects of labeling time and HU on replication fork asymmetry.

(**A**) Summary of fork asymmetry analyses shown in Fig. 2B ($n = 3$ biological replicates). Data are presented as mean +/− SD of the % asymmetric forks (percentage of forks in the asymmetric region indicated in Fig. 2A) in the repeated experiments. (**B**) Fork asymmetry rate in ES cells with longer pulse-labeling times (IdU - 30 min and CldU - 30 min) (= 30-30, L) and compared with standard labeling (15-15, S). $n = 3$ independent experiments. Data are presented as mean +/− SD. (**C**) Fork asymmetry rate in low (10 μM) and high (100 μM) concentrations of HU. $n = 3$ independent experiments. NS, not significant ($P \geqq 0.05$), *$P < 0.05$, **$P < 0.01$, two-tailed *t* tests. Exact *P* values are also indicated. At least 50 DNA fibers are scored to evaluate fork asymmetry rate per experiment.

 © The Author(s)

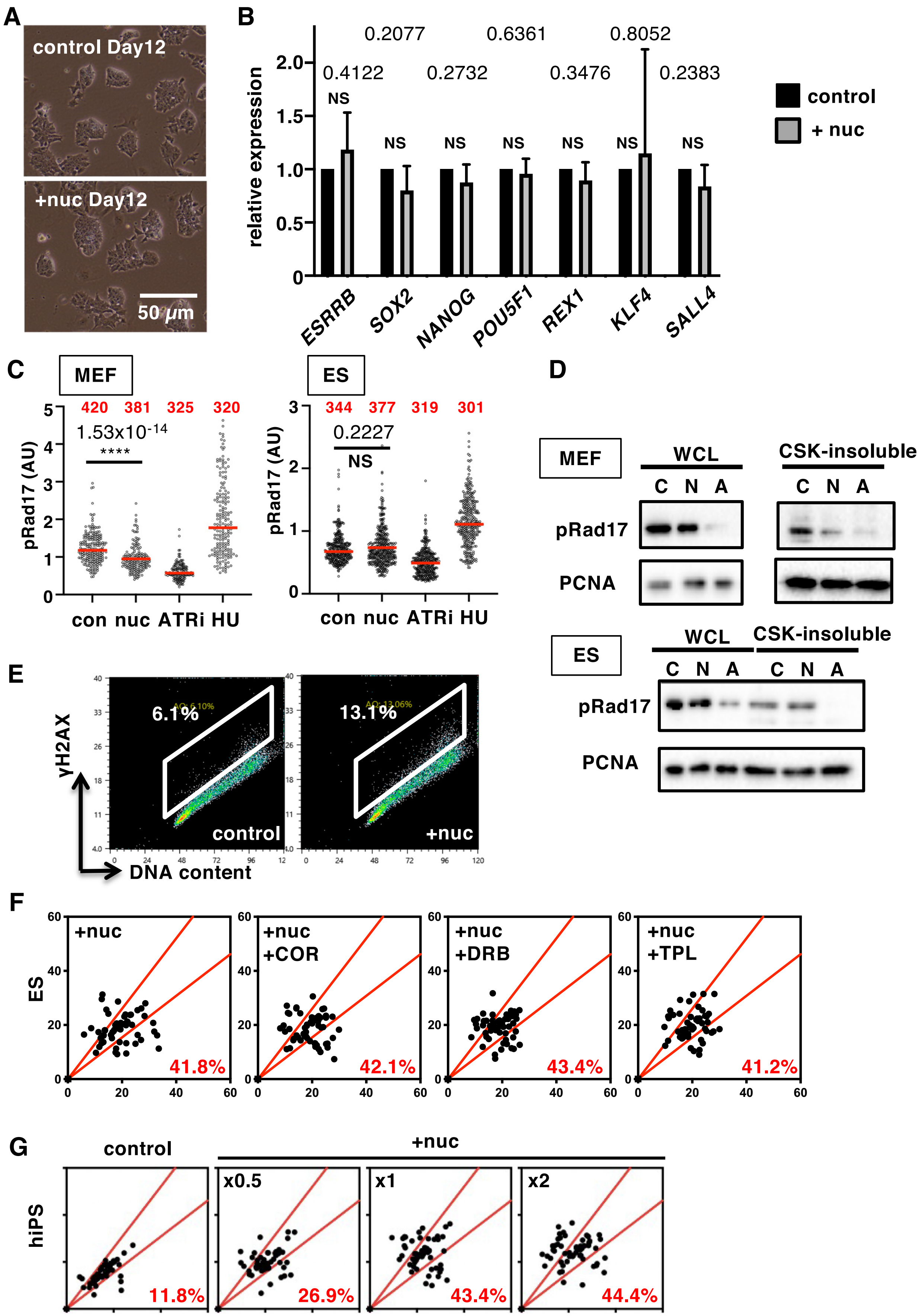
A
control Day12
+nuc Day12
50 μm
B
relative expression
0.4122
0.2077
0.2732
0.6361
0.3476
0.8052
0.2383
NS
control
+ nuc
ESRRB
SOX2
NANOG
POU5F1
REX1
KLF4
SALL4
C
MEF
ES
pRad17 (AU)
420 381 325 320
1.53x10-14
****
344 377 319 301
0.2227
NS
con nuc ATRi HU
D
MEF
WCL
CSK-insoluble
C N A
pRad17
PCNA
ES
E
γH2AX
DNA content
6.1%
13.1%
control
+nuc
F
ES
+nuc
+nuc +COR
+nuc +DRB
+nuc +TPL
41.8%
42.1%
43.4%
41.2%
G
control
+nuc
x0.5
x1
x2
hiPS
11.8%
26.9%
43.4%
44.4%

© The Author(s)

Figure EV3. Effects of nucleoside addition.

(**A**, **B**) ES cells were cultured with (+nuc) or without (control) nucleosides for 12 days with daily media changes, and their morphology (A) and pluripotency-associated gene expression (**B**: $n = 3$ biological replicates, each with 2 technical replicates) were evaluated. Data presented as mean +/− SD. NS, not significant ($P \geqq 0.05$), two-tailed *t* tests. (**C**) MEF and ES cells were treated with nucleosides (48 h), 1 µM ATRi (2 h) or 1 mM HU (2 h) prior to fixation and immuno-staining of the phosphorylated form of Rad17 (pRad17) was carried out as a measure of ATR activity. Signal intensities were quantitated for plots shown in Fig. 3D, but only within S phase which includes all the data points in between left and right borders (including points outside box, towards bottom). The number of cells scored are indicated in red. Red bars indicate mean. NS, not significant ($P \geqq 0.05$), ****$P < 0.0001$, Mann–Whitney tests. Exact *p* values are also indicated. (**D**) MEF and ES cells were treated with (N) or without (**C**) nucleosides or 1 µM ATRi (A) for 2 h and were collected for western blot analysis. WCL; whole-cell lysate, CSK-insoluble; Cytoskeleton buffer-insoluble (= nuclear fractions). PCNA is used as an internal control. (**E**) ES cells grown in normal condition (control) or with nucleosides for 48 h were fixed and stained with anti-γH2AX antibodies and a DNA binding dye (FxCycle Violet), and were subjected to flow cytometry. (**F**) Fork asymmetry analyses were carried out using ES cells treated with nucleosides for 48 h with or without transcription inhibitors (COR, DRB, TPL) for 2.5 h as in Fig. EV1B. (**G**) Human iPS cells were treated with different concentrations of nucleosides (0.5×, 1×, or 2× suggested concentrations) for 72 h and were collected for DNA fiber assay and fork asymmetry analyses. Data from a representative experiment is shown ($n = 2$ independent experiments) for (**C–G**).

 © The Author(s)

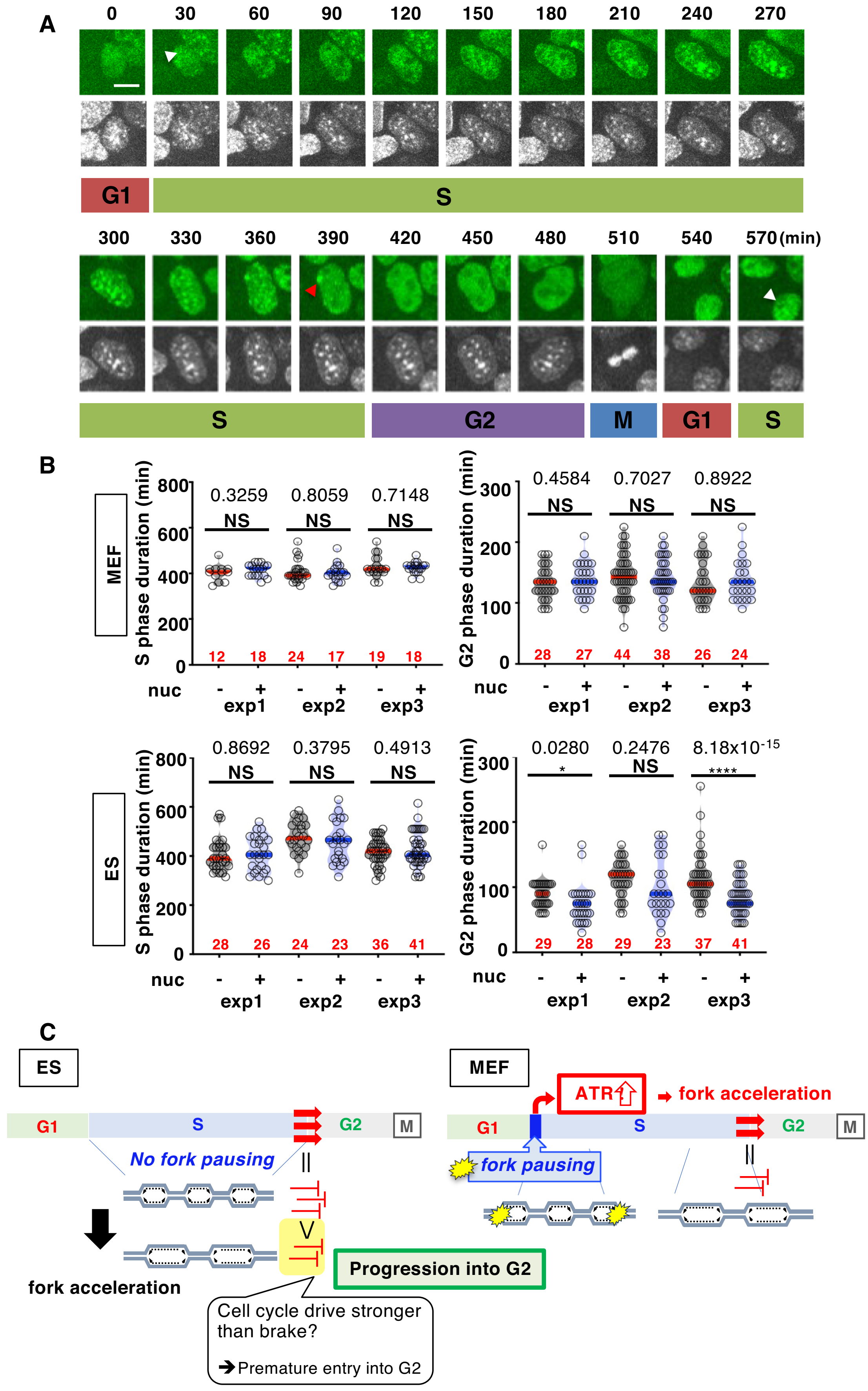
A
0 30 60 90 120 150 180 210 240 270
G1
S
300 330 360 390 420 450 480 510 540 570 (min)
S
G2
M
G1
S
B
MEF
S phase duration (min)
0.3259 0.8059 0.7148
NS NS NS
12 18 24 17 19 18
nuc - + - + - +
exp1 exp2 exp3
G2 phase duration (min)
0.4584 0.7027 0.8922
NS NS NS
28 27 44 38 26 24
ES
0.8692 0.3795 0.4913
NS NS NS
28 26 24 23 36 41
0.0280 0.2476 8.18x10-15
* NS ****
29 28 29 23 37 41
C
ES
G1 S G2 M
No fork pausing
fork acceleration
Progression into G2
Cell cycle drive stronger than brake?
Premature entry into G2
MEF
ATR
fork acceleration
fork pausing

Figure EV4. Measurement of cell cycle duration and the model.

(**A**) An example of time-lapse images of PCNA-mNG used in Fig. 4A and Fig. EV4B. The time at which the imaged frame exhibited PCNA foci (white arrowhead) was defined as the start of S phase and that of PCNA foci disappearance (red arrowhead being the last frame before disappearance) was defined as the end of S phase. The time at which PCNA signal spread out to the cytoplasm as a result of nuclear membrane breakdown, was defined as mitosis. Chromosome condensation is also evident at this point. Bar, 10 μm. (**B**) Repeat experiments shown in Fig. 4A. Exp1 is the same as the data shown in Fig. 4A. NS, not significant ($P \geqq 0.05$), *$P < 0.05$, ****$P < 0.0001$. Two-tailed *t* tests. Exact *P* values are also indicated. The numbers of scored cells are indicated in red. (**C**) A proposed model. See main text for details.

© The Author(s)
